# REIMAGINING ADVANCE CARE PLANNING: FORMER CAREGIVERS OF AFRICAN AMERICANS WITH DEMENTIA PERSPECTIVES

**DOI:** 10.1093/geroni/igac059.841

**Published:** 2022-12-20

**Authors:** Emika Miller, Karen Moss, Kathy Wright, Kimberly Wilson-Lawson, Mary Beth Happ, Karen Rose, Todd Monroe, Celia Wills

**Affiliations:** Ohio State University, Columbus, Ohio, United States; The Ohio State University, Columbus, Ohio, United States; The Ohio State University College of Nursing, Columbus, Ohio, United States; African American Alzheimer's and Wellness Association, Columbus, Ohio, United States; The Ohio State University, Columbus, Ohio, United States; The Ohio State University, Columbus, Ohio, United States; Ohio State University College of Nursing, Columbus, Ohio, United States; Ohio State University College of Nursing, Columbus, Ohio, United States

## Abstract

Advance care planning disparities persist among lower socioeconomic status African American older adults living with dementia despite multiple decades of research. The perspectives of former family caregivers are rarely used in developing effective interventions. This study aims to characterize the advance care planning and other health-related decision-making perspectives of former family caregivers of African Americans who lived with dementia. This qualitative study is part of an ongoing mixed-methods study to co-create a culturally tailored advance care planning intervention. Former caregivers (n=11) participated in semi-structured interviews and identified the following needs: Caregiver education, caregiver recognition by healthcare providers, and removal of caregiving obstacles. Participants also acknowledged their need to restore identity following the death of their care recipient and a desire to assist other caregivers through research participation. This study fills a critical gap by including former caregivers’ perspectives in co-creating a culturally tailored advance care planning intervention.

